# TNF-α Expression and Plasma Cell and Macrophage Numbers in Traumatic Ulcers in a Rat Model Treated by Topical or Systemic Probiotic *L. casei Shirota*

**DOI:** 10.7150/ijms.99156

**Published:** 2025-01-01

**Authors:** Irma Josefina Savitri, Safira Alif Suprarahardiani, Laila Kamilatu Ulinnuha, Chiquita Prahasanti, I Komang Evan Wijaksana, Rini Devijanti Ridwan, Diah Savitri E, Mohammed Ahmed Aljunaid, Sadatullah Syed, Shahabe Saquib Abullais

**Affiliations:** 1 Department of Periodontology, Faculty of Dental Medicine, Universitas Airlangga Surabaya, Indonesia.; 2 Research Center for Biomaterial and Tissue Engineering, Universitas Airlangga Surabaya, Indonesia.; 3 Dental Medicine, Faculty of Dental Medicine, Universitas Airlangga Surabaya, Indonesia.; 4 Department of Oral Biology, Faculty of Dental Medicine, Universitas Airlangga Surabaya, Indonesia.; 5 Department of Oral Medicine, Faculty of Dental Medicine, Universitas Airlangga Surabaya, Indonesia.; 6 Lectures in Department of Oral and Dental Medicine, Faculty of Medicine, Taiz University, Taiz-Yemen.; 7 Diagnostic Dental Sciences, College of Dentistry, King Khalid University, 62529 Abha, Saudi Arabia.; 8 Central Labs, King Khalid University, AlQura'a, Abha, P.O. Box 960, Saudi Arabia.; 9 Department of Periodontics and Community Dental Sciences, College of Dentistry, King Khalid University, 62529 Abha, Saudi Arabia.

**Keywords:** *L. casei Shirota*, TNF-α expression macrophages, plasma cells, healthy lifestyle

## Abstract

**Introduction**: Live microorganisms, named probiotics, can improve overall physical well-being, particularly the oral cavity's health. *L. casei Shirota*, a popular probiotic, can influence the immune response by increasing the number of macrophages and plasma cells that play a role in traumatic ulcer healing.

**Aims**: To determine the expression of tumor necrosis factor-alpha (TNF-α) and the varied number of plasma cells and macrophages on a traumatic ulcer animal model treated with topical or systemic administration of a probiotic *L. casei Shirota.*

**Material and Methods**: Thirty-six healthy, 2-3-month-old male *Rattus norvegicus* weighing 175-250 gram, were designed into control and topical and systemic administration probiotic groups. The control group was treated with Aquadest 20 uL/20 gr, while topical probiotic and systemic administration probiotic groups were treated with 10,9x10^7^ cells/kg, respectively. A heated round burnisher tip was used to traumatize the inferior incisive fornix labial area. After 3 or 7 days, the animal models were terminated. Immunohistochemical examination, Hematoxylin eosin staining, and statistical analysis were performed to analyze the expression of TNF-α and the number of plasma cells and macrophages.

**Results**: The Mann-Whitney and Tukey HSD tests indicated significant differences (*p* < 0.05) in the results for the three groups. It was observed that topical administration provides more remarkable results than systemic administration for the expression of TNF-α, the number of plasma cells, and the number of macrophages.

**Conclusion**: Topical administration of *L. casei Shirota* demonstrates better results than systemic administration for healing traumatic ulcers.

## Introduction

Wound healing is a complex and dynamic process that plays a crucial role in maintaining tissue integrity following injury. Traumatic ulcers, which are common oral mucosal lesions, present significant clinical challenges due to their potential for discomfort, infection, and delayed healing 1,2. The management of these ulcers typically involves conventional therapies such as topical steroids, antiseptics, and antibiotics, which, while effective, may lead to complications like antibiotic resistance and adverse reactions 3. Given these limitations, there is a growing interest in exploring alternative therapeutic approaches, such as the use of probiotics, which are live microorganisms that confer health benefits to the host when administered in adequate amounts 4.

Probiotics, particularly strains of *Lactobacillus*, have been extensively studied for their beneficial effects on the immune system and their potential to modulate inflammatory responses 5,6. *L. casei Shirota*, a commonly used probiotic, has exhibited promise in altering gut microbiota and influencing systemic immune responses, including regulating pro-inflammatory cytokines and promoting tissue healing. The therapeutic potential of probiotics extends beyond gut health, with emerging evidence suggesting that they can enhance wound healing, reduce inflammation, and improve outcomes in various inflammatory conditions, including oral ulcers 7.

Probiotics are increasingly being recognized for their potential therapeutic applications in wound healing and inflammation modulation 5,8. Oral wounds resulting from surgeries, traumatic injuries, or chronic conditions present considerable clinical challenges due to the risk of infection, prolonged inflammation, and delayed healing. Emerging research suggests that probiotics can be crucial in addressing these challenges by enhancing the healing process and reducing inflammation. Campos *et al.* reported that probiotics accelerated wound healing and increased collagen deposition in diabetic rats, highlighting their potential to modulate the inflammatory response to enhance healing 7.

The therapeutic potential of probiotics in wound healing extends beyond simple acceleration of the healing process. Various studies have indicated that probiotics can reduce inflammation, which is critical for optimal wound recovery. Tagliari *et al.* discovered that probiotics shortened the inflammatory phase and improved healing outcomes by reducing levels of pro-inflammatory cytokines such as Interleukin (IL)-6, tumor necrosis factor-alpha (TNF-α), and IL-17 in rats 9. Clinical applications are equally promising; Twetman *et al.* reported a tendency for improved oral wound healing in participants using *Lactobacillus reuteri* lozenges, marked by higher expressions of TNF and IL-8, suggesting an enhanced inflammatory response aiding wound healing 10. Additionally, Marlina *et al.* observed that VSL#3 probiotics reduced pro-inflammatory cytokines and accelerated wound healing in patients with oral lichen planus 11. These findings are further supported by systematic reviews such as one by Togo *et al.*; they highlighted the general improvement in wound healing outcomes with probiotic use and the absence of adverse effects, suggesting a strong potential for clinical applications 12. Probiotics also exhibit antagonistic activity against pathogens; Fijan *et al.* indicated their role in preventing infections and promoting wound healing through enhanced immune response 13. Innovative approaches, such as self-healing probiotic-loaded hydrogels developed by Mei *et al.*, have shown significant promise in inhibiting infection and inflammation, further underscoring the multifaceted benefits of probiotics in wound management 14. Overall, the cumulative evidence strongly supports the incorporation of probiotics as a valuable adjunctive therapy in oral wound healing and inflammatory modulation.

The healing process of oral traumatic ulcers involves a coordinated interplay between immune cells, cytokines, and other mediators of inflammation and tissue repair 15,16. Macrophages, which are crucial in the early and late stages of wound healing, undergo phenotypic changes that drive the transition from inflammation to tissue repair 17,18. Similarly, plasma cells play a critical role in the immune response by producing antibodies that help eliminate pathogens and regulate inflammation 19,20. Since these cells are vital in wound healing, it is essential to understand how probiotic treatment affects their activity and abundance during the healing of traumatic ulcers. However, the specific effects of *L. casei Shirota*, particularly when applied topically versus systemically, on the healing of traumatic ulcers remain underexplored.

Despite the growing body of evidence supporting the use of probiotics in wound healing, there remains a significant gap in understanding the differential effects of topical versus systemic administration of probiotics on oral traumatic ulcers. Given the distinct mechanisms through which probiotics exert their effects, it is essential to investigate how these different delivery modes influence critical aspects of wound healing, such as the expression of pro-inflammatory cytokines like TNF-α and the involvement of immune cells, including plasma cells and macrophages.

The objective of this study is to investigate the effects of *L. casei Shirota*, administered either topically or systemically, on the expression of TNF-α and the number of plasma cells and macrophages in a rat model of traumatic ulcers. This research aims to fill the knowledge gap by comparing the impact of different probiotic delivery methods on key inflammatory and immune parameters involved in ulcer healing. By elucidating these effects, this study seeks to contribute to developing more effective probiotic-based therapies for managing traumatic ulcers, offering a potential alternative to conventional treatments with significant side effects. The novelty of this research lies in its focus on the differential outcomes of topical versus systemic probiotic administration, which has not been thoroughly explored in the context of traumatic ulcer healing.

## Material and Methods

### Oral traumatic ulcer animal model preparation

**Figure 1:**
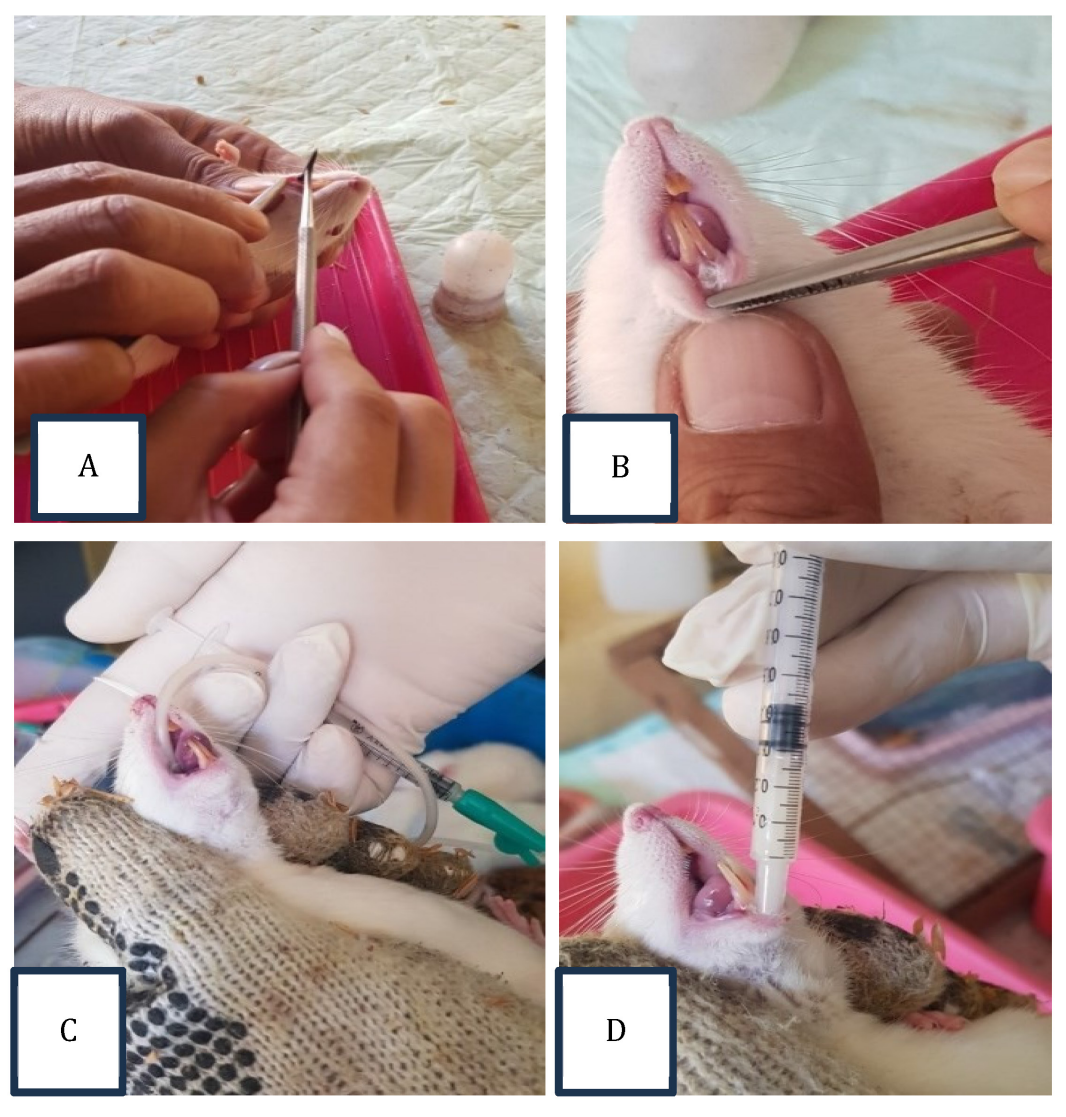
The procedure of oral traumatic ulcer animal model preparation**. (**A) The heated round burnisher's tip with a 2 mm diameter was placed on the mandibular labial mucosa for 15 seconds. (B) The oral traumatic ulcers formed 24-48 hours after this. (C) *L. casei Shirota* (1.09 ml/kg body weight) was fed directly to the gastrointestinal tract using a feeding tube. This procedure was designed for the systemic probiotic administration treatment groups. (D) *L. casei Shirota* (1.09 ml/kg body weight) was applied topically on the traumatic ulcers using a Spuit 1 cc tuberculin for the topical probiotic administration treatment groups.

The Committee of Dental Medicine, Universitas Airlangga, Indonesia, has granted ethical approval for this research (No. 335/HRECC. FODM/VII/2020). Thirty-six healthy, 2-3-month-old male *Rattus norvegicus* weighing 175-250 grams were designed into control and systemic and topical administration probiotic treatment groups. A heated, round burnisher's tip was used to create a traumatic ulcer with a diameter of 2 mm under an anesthesia procedure using Ketamin HCL 50 mg/ml (OGB dexa, Indonesia); the tip was heated for 15 seconds, then touched for 1 second on the inferior incisive fornix labial (mandibular labial mucosa) area of the experimental animal to a depth equal to the diameter of the burnisher's tip. The ulcers occurred 24-48 hours post-wound (Fig. 1A-B) 4,21.

**Figure 2:**
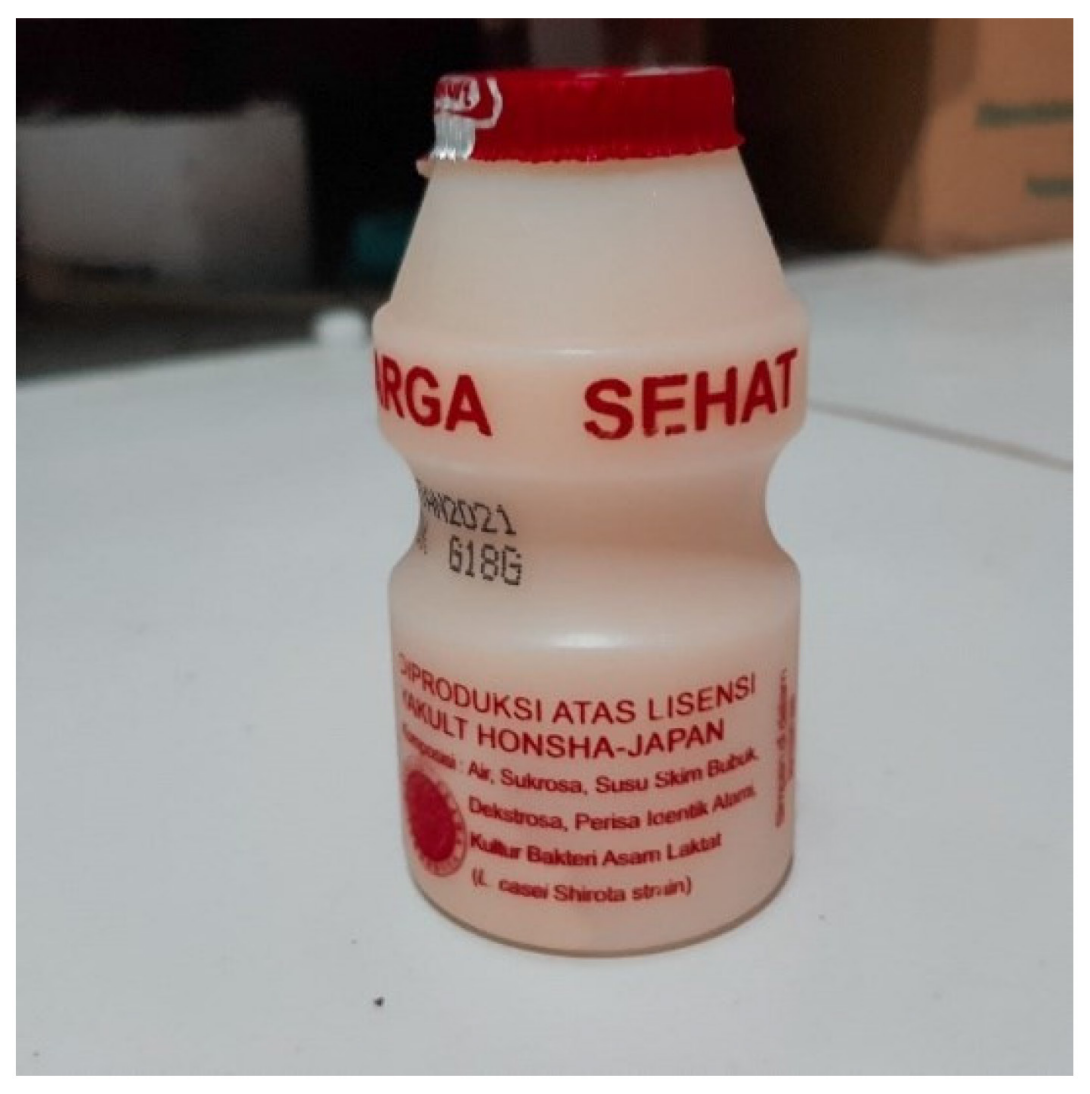
Yakult Persada Indonesia, a product containing 6.5 x 10^9^* L. casei Shirota*.

In the control group, the treatment was undertaken by topically dropping the distilled water on the traumatic ulcers in a dose of 20 µL/20 g body weight every day for 3 or 7 days. *L. casei Shirota* (Yakult Indonesia Persada) (Fig. 2) was used in topical and systemic administration probiotic treatment groups. In the systemic administration treatment group, *L. casei Shirota* —10.9 x 10^7^ cells/kg body weight (1.09 ml/kg body weight) —was fed directly to the gastrointestinal tract using a feeding tube every day for 3 or 7 days. In the topical administration treatment group, *L. casei Shirota* was applied topically, dropping as much as 10.9 x 10^7^ cells/kg body weight (1.09 ml/kg) on the traumatic ulcer every day for 3 or 7 days. The application was conducted once a day until the termination procedure on 3 or 7 days (Fig. 1C-D) 21.

### Paraffin Block Procedure

The fixation with 10% neutral buffered formalin solution was performed; the tissue was soaked at least once for 24 hours in a solution of volume ten times the size of the specimen. The dehydration process was started by applying alcohol, 70% and 80% each, for 1 hour, 90% for 1 hour twice, and 100% for 1 hour thrice. The process was continued by clearing steps using xylol three times for 1 hour, 2 hours, and 3 hours. The impregnation process (paraffin infiltration process) was performed by melting solid paraffin at 60⁰C and inserting the tissue into the paraffin. Embedding tissue in solid paraffin to obtain a paraffin block was the last step of the procedure 22.

### Immunohistochemical Examination

The immunohistochemical examinations were performed using the manufacturers' protocols. Briefly, the slides were deparaffinized with xylol 1, 2, and 3 solutions for 5 minutes each. Then, the rehydration process was conducted using a solution of 96% alcohol, 80% alcohol, and 70% alcohol; the slides were soaked in running water for 5 minutes and dried for 15 minutes. After drying, the slides were placed in a Tris Buffered Saline (TBS) pH 7.4 solution for 5 minutes and blocked with peroxidase for 5 to 10 minutes. The incubation process was performed with TNF-α anti-rat monoclonal antibody (Biogearscientific), after which the slides were washed in a TBS pH 7.4 solution for 5 minutes and stored in the rack for 30 minutes.

The slides were then washed in a TBS pH 7.4 solution for 5 to 10 minutes. After washing, a Diamino Benzidine (DAB) chromogen + Substrate Chromogen (in a ratio of 20µL:1000µL) solution was dropped on the slides for 5 minutes. The slides were rinsed with running water and stained with Hematoxylin. The preparations were washed with running water for 5 minutes; subsequently, the slide preparations were dipped in lithium carbonate for 2 minutes, re-washed with running water, and dried with 80% and 96% alcohol, respectively, for 5 minutes, and cleared with an aqueous solution of xylol 1, 2, and 3 for 5 minutes each. The slide preparations were covered with a cover glass and ready for calculation.

### Hematoxylin Eosin Staining

Coloring tissue preparations began with deparaffinization resistance using xylol. The preparation was kept in xylol for 2 minutes, and the process was repeated for 2 minutes in another container. The preparation was dehydrated with 96%, 95%, and 80% alcohol each for 1 minute. The preparation was rinsed under running water for 10-15 minutes, from a slow stream to a strong stream, to remove all excess alcohol. The preparation was stained with Mayer's Hematoxylin dye for 15 minutes and rinsed again with running water for 20 minutes. The preparation was soaked in eosin from 15 seconds to 2 minutes and dehydrated with alcohol solution at higher concentrations of 95% and 96% each for 2 minutes, twice in different containers. After going through the absolute alcohol, the preparation was transferred to xylol and mounted. The Entellan medium was dropped on the glass refractive index in the smear preparation. The preparation was covered with a cover glass and dried.

Using a light microscope, the number of cells that expressed TNF-α, macrophage and plasma cells taken from the mandibular labial mucosa were observed and calculated with 400x magnification at five fields of view. The ANOVA test was used to analyze the collected data, and a *p*-value of ≤ 0.05 was considered statistically significant.

## Results

Clinical appearance in the form of traumatic ulcers was obtained in the animal model. The healing process of the wound occurred after systemic or topical administration of probiotic treatment. Optimal recovery was shown by topical administration procedure after 7 days of treatment.

A 400x light microscope was used to determine TNF-α expression and calculate the quantity of macrophages and plasma cells. The Kolmogorov-Smirnov test's normality checks revealed that all research groups had normally distributed data (*p*-value> 0.05). Levene's test results revealed that all sample groups were homogeneous (*p*-value> 0.05); the results in each sample group were compared using ANOVA one-way test findings with post hoc Tukey HSD.

**Figure 4:**
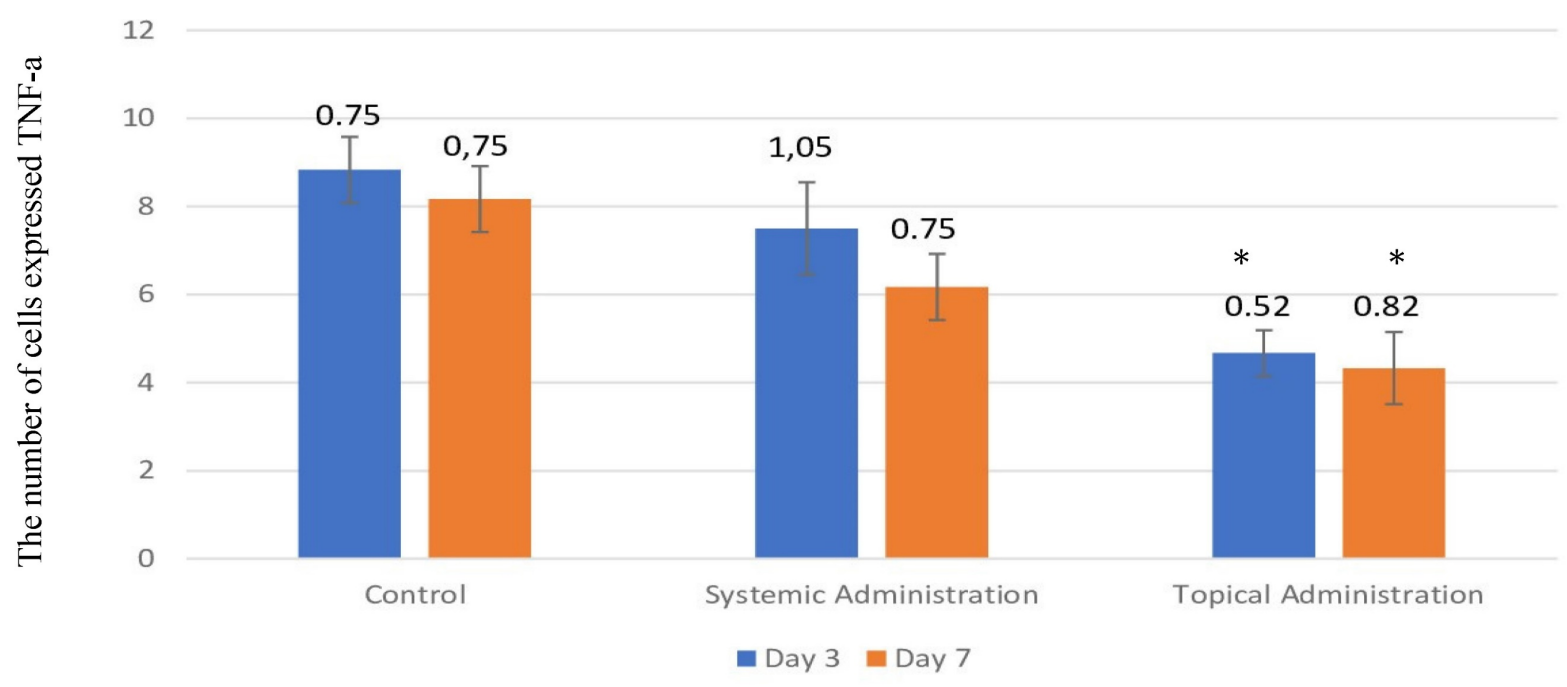
The expression of TNF-α after 3 or 7 days of treatment with 10.9 x 10^7^ cells/kg of* L. casei Shirota* in systemic or topical administration. *p* < 0.05.

**Figure 5:**
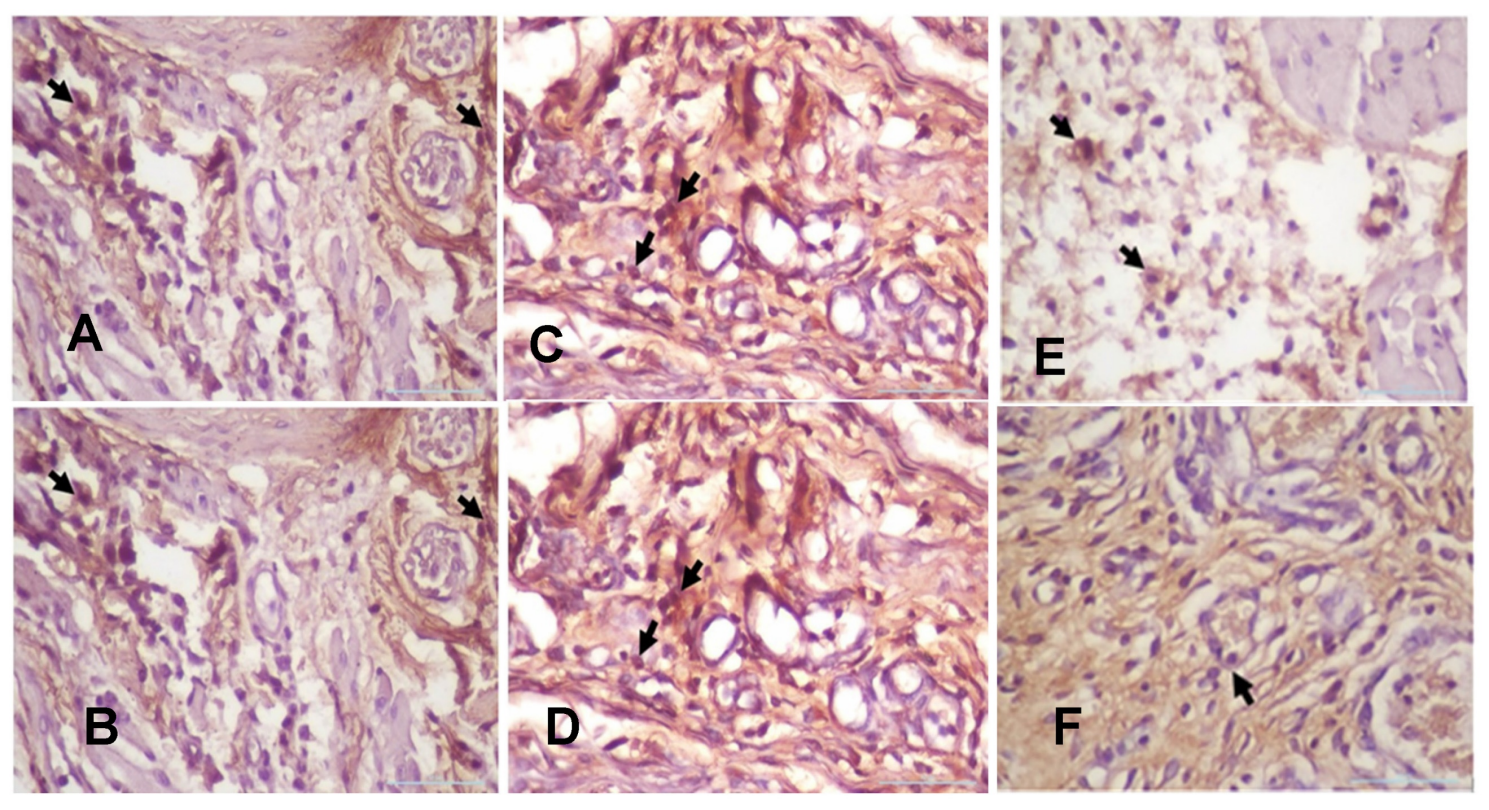
(A) The 3-day control group's TNF-α expression. (B) The 7-day control group's TNF-α expression. (C) The 3-day systemic group's TNF-α expression (D). The 7-day systemic group's TNF-α expression. (E) The 3-day topical group's TNF-α expression. (F) The 7-day topical group's TNF-α expression. *p* < 0.05.

The number of cells that expressed TNF-α in the 3-day control group was present in the highest quantity in the oral traumatic ulcer animal model (Fig. 4 and Fig. 5A-B); the mean of the 3-day control group was 8.33. However, the *L. casei Shirota* probiotic treatment decreased the expression of TNF-α. The topical administration significantly decreased TNF-α after 3- or 7-day treatment (Fig. 5E-F).

**Figure 6:**
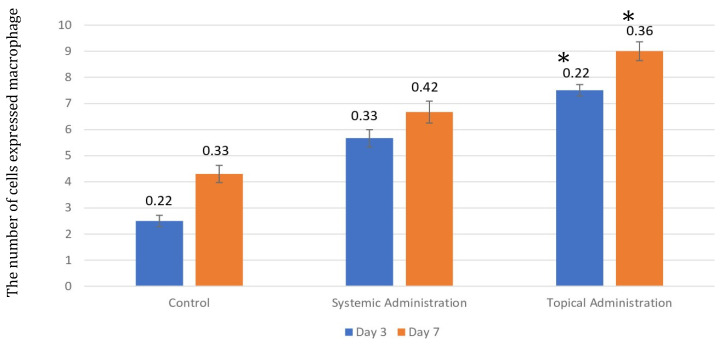
The number of macrophages after 3 and 7 days of treatment by 10.9 x 10^7^ cells/kg of* L. casei Shirota* in systemic and topical administration. *p* < 0.05.

**Figure 7:**
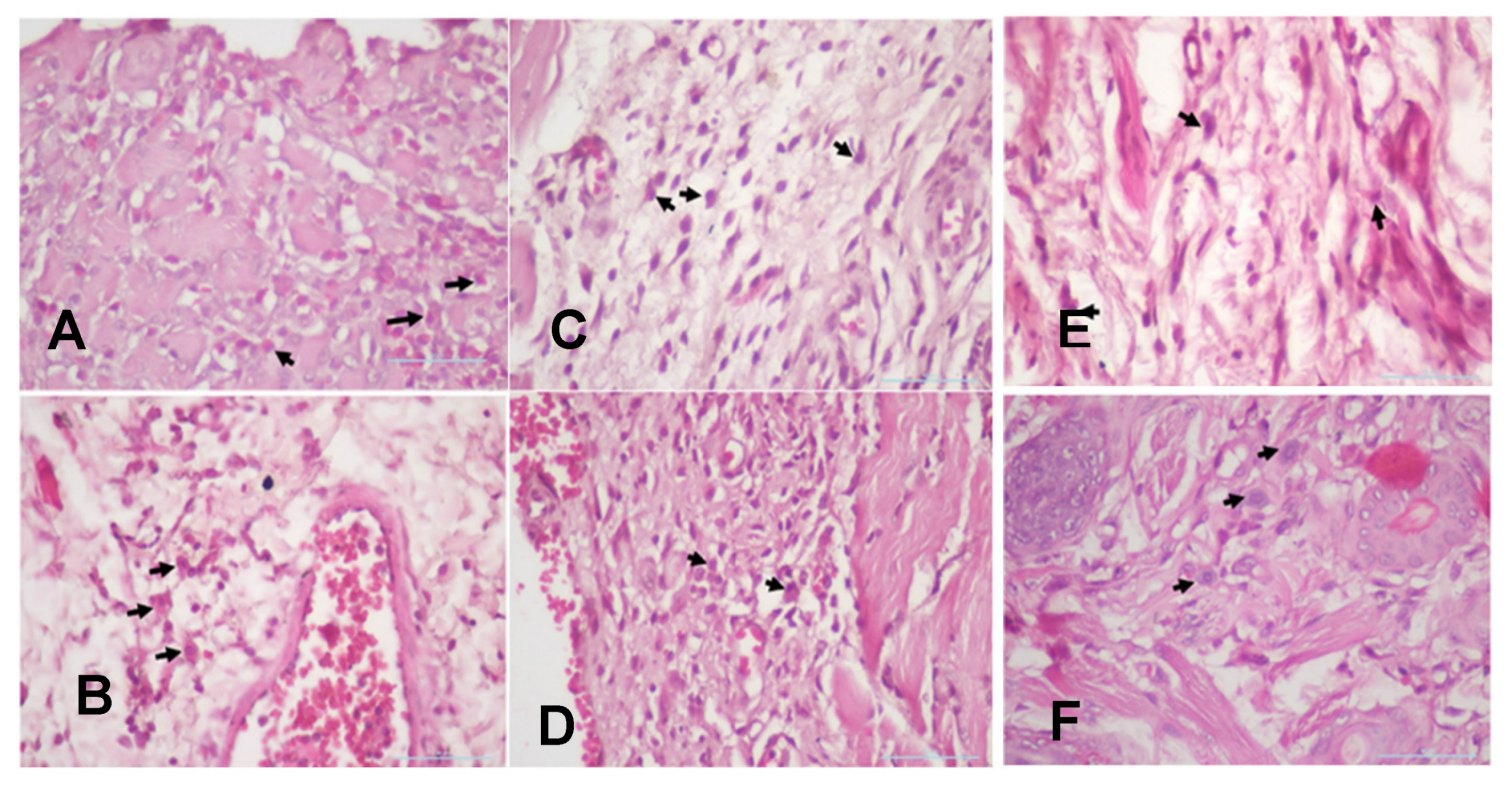
(A). The 3-day control group's macrophages. (B) The 7-day control group's macrophages. (C). The 3-day systemic group's macrophages. (D). The 7-day systemic group's macrophages. (E). The 3-day topical group's macrophages (F). The 7-day topical group's macrophages.

**Figure 8:**
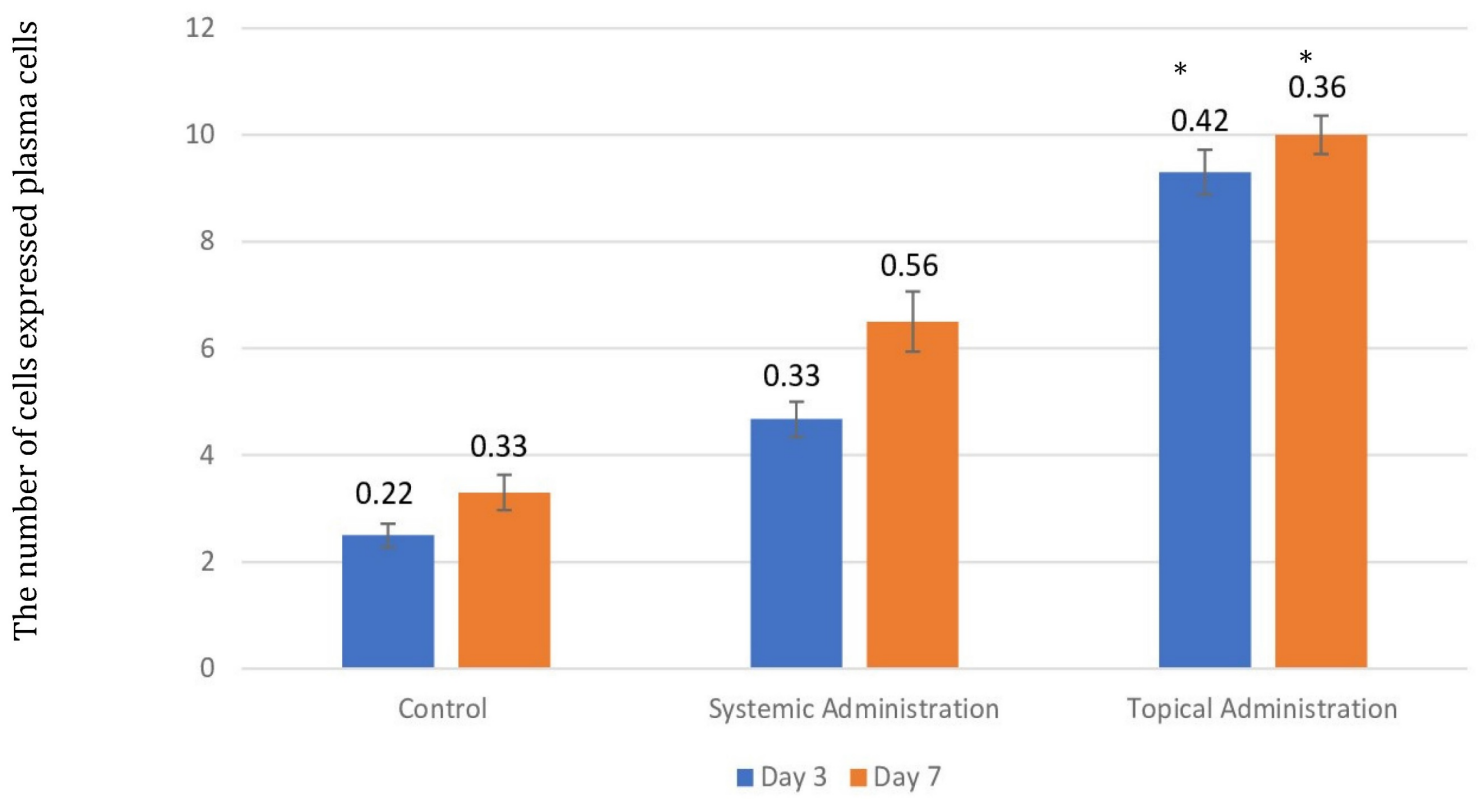
The number of plasma cells after 3 and 7 days of treatment with 10.9 x 10^7^ cells/kg of* L. casei Shirota* in systemic and topical administration groups. *p* < 0.05.

**Figure 9:**
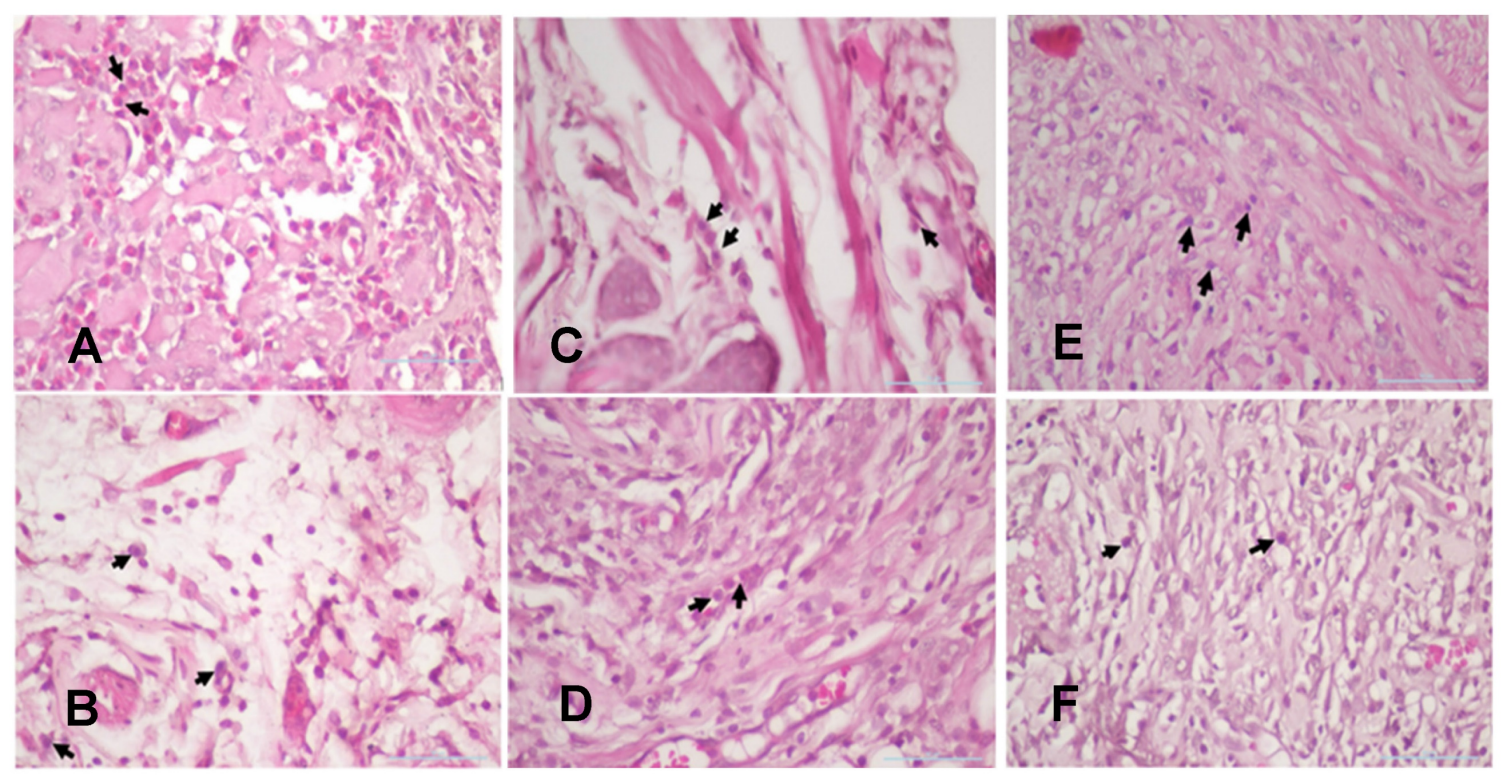
(A). The 3-day control group's plasma cells (B). The 7-day control group's plasma cells (C). The 3-day systemic group's plasma cells (D). The 7-day systemic group's plasma cells (E). The 3-day topical group's plasma cells. (F). The 7-day topical group's plasma cells.

The injury on mandibular labial mucosa caused the ulceration and triggered the inflammation. The inflammation is marked by the infiltration of macrophages (Fig.6 and Fig. 7A-B) and plasma cells (Fig. 8 and Fig. 9A-C). The *L. casei Shirota* probiotic treatment recovered a significant number of macrophage and plasma cells compared with the control group. This number was twice as high for the topical probiotic administration group compared to the systemic administration treatment group for 3 and 7 days.

## Discussion

The current study provides compelling evidence that topical administration of *L. casei Shirota* significantly enhances the healing process of traumatic ulcers. This finding is particularly relevant in the context of existing literature, which has increasingly recognized the therapeutic potential of probiotics in wound care 7,9, but has not extensively explored the comparative efficacy of different administration routes.

The enhanced wound healing observed with topical probiotics, as evidenced by the marked increase in macrophages and plasma cells and the reduction of TNF-α expression, suggests a potent localized effect. This outcome highlights the ability of probiotics to modulate the immune response directly at the site of injury, offering a targeted approach that systemic administration may not fully replicate. These findings align with earlier research indicating that probiotics can reduce inflammation and promote tissue repair 5,6,9; however, this study expands upon these insights by demonstrating that the route of administration plays a critical role in determining the efficacy of the treatment.

Probiotics reduce inflammation by lowering the activity of dendritic cells, resulting in a decrease in the number of CD4 + T-Cells, reducing the production of pro-inflammatory cytokines (IL-1, IL-6, TNF-α, INF-γ) and high sensitivity C reactive protein. Probiotics increase the production of pro-inflammatory cytokines (IL-10 and IL-4); in inflamed mucosa, probiotics inhibit TNF-α production. In addition, probiotics regulate lymphocyte proliferation, phagocytosis, serum IgA, and the severity of atopic dermatitis. Immunomodulation reduces susceptibility to inflammatory and allergic factors. Systemic administration of probiotics can increase the body's immunity 7,9.

In this study, the TNF-α expression was carried out after administering the probiotic *L. casei Shirota* for 3 days or 7 days, and the number of macrophages and plasma cells was increased in the topical and systemic treatment groups compared with the control group. In 2019, Tagliari *et al.* discovered that topical probiotic treatment could improve wound healing by decreasing bacteria and increasing tissue repair in rat wounds (Wistar rats) 9. In traumatic ulcer therapy, topical and systemic medication is required, which not only lessens pain (a symptom) but also accelerates the healing process 23. According to this research, the quantity of macrophages and plasma cells in the topical therapy group was 7 days greater than in the other groups.

TNF-α is a pro-inflammatory cytokine produced by macrophages. TNF-α functions to stimulate inflammatory cells, fibroblasts, and epithelium. When there is a wound present, TNF-α levels increase. The higher TNF-α level in the wound indicates an ongoing inflammatory process. An increase in TNF-α levels can induce the release of the endothelial adhesion molecule, namely intercellular 3 adhesion molecule 1 (ICAM-1), which increases the attachment of neutrophils to endothelial cells before entering the intracellular space. Other inflammatory products cause neutrophil chemotaxis toward injured tissue. Probiotics can inhibit TNF-α production in inflamed mucosa 19,20. Probiotics cause increased proliferation of immune cells and reduce the production of pro-inflammatory cytokines such as TNF-α and IL-6. In addition, probiotics suppress lymphocyte proliferation and the production of pro-inflammatory cytokines by T-cells. The immunomodulatory effects of probiotics can reduce the severity of atopic dermatitis by inhibiting Th2 cell responses, cytokines such as IL-4, IL-5, IL-6, and IL-13 are no longer secreted, INF-γ (cytokines released by Th1 cells) is decreased, phagocytosis is stimulated, and serum IgA increases. Probiotics also stimulate the secretion of IL-10 and Transforming Growth Factor-β (TGF-β). Systemic administration of probiotics can increase the body's immunity, as probiotics interact with antigen-presenting cells (macrophages and dendritic cells) 4,5,12,18.

This study's results also suggest that the localized action of probiotics through topical application may offer distinct advantages over systemic delivery. The direct interaction with the wound environment allows probiotics to exert their effects more rapidly and effectively, potentially leading to faster and more complete healing. This contrasts with systemic administration, where the probiotics must first be processed through the body's various systems before reaching the target area, potentially diluting their efficacy 24. This distinction is crucial for developing more efficient wound care strategies, particularly in clinical settings where rapid recovery is essential.

Probiotics may be more beneficial for the wound healing process when applied topically. This is in line with some research, which found that topical probiotic administration is a more effective treatment than systemic probiotic administration because, in topical administration, a combination of mechanisms between probiotic bacteria intervention and immune system stimulation is beneficial for the healing process. Additionally, probiotics applied topically naturally enter the digestive system, enabling interactions between lymphoid tissue and probiotic bacteria there. Additional impacts on the wound healing process are seen due to immune system modification 8. Probiotics interact with antigen-presenting cells (macrophages and dendritic cells). The interactions carried out by probiotics will cause macrophages and dendritic cells to release several chemical mediators, such as cytokines, which regulate the function of Treg cells to induce immunomodulation of the body. So probiotics in the wound area can speed up the healing process because they can increase the number of plasma cells and macrophages and reduce the production of TNF-α 24,25.

Furthermore, the observed increase in immune cell activity, particularly the proliferation of macrophages and plasma cells, provides a mechanistic understanding of how topical probiotics may accelerate wound healing. Macrophages play a key role in the early stages of wound healing by clearing debris and pathogens, while plasma cells contribute to the immune response by producing antibodies. The enhancement of these processes through topical probiotic administration suggests that probiotics modulate the immune response and actively support the physiological processes required for tissue repair. These findings build upon previous studies that have identified probiotics as modulators of immune function, but they extend this knowledge by detailing how these effects manifest in the specific context of wound healing 20.

Macrophages will appear in the wound area and continue the process of phagocytosis 48 to 72 hours after the injury. Macrophages are attracted to the wound area by myriad inflammatory mediators such as clotting factors, complement components, and cytokines such as PDGF, TGF-β, leukotriene B4, and platelet factor IV. Macrophages will secrete factors of growth, especially TGF-β, as well as other mediators (TGF-α, factors heparin-binding epidermal growth, fibroblast growth factor [FGF], collagenase), activating keratinocytes, fibroblasts, and endothelial cells. Macrophages, which are larger and have stronger phagocytic abilities, usually follow and play a role in clearing pathogenic remains and dead cells and stimulating the healing process 19,20.

In all tissues, macrophages work as filters for causative particle problems, microbes, and senescent cells, which work like sentinels to alert specific components of the adaptive immune system (T lymphocytes and B) to stimulate injury. Plasma cells develop B lymphocytes, are activated, and produce antibodies to fight persistent antigens in areas of inflammation or against tissue components that change. The timing of plasma cells appearing in an inflammatory response can vary depending on the type and extent of inflammation and the individual's immune system. In many cases, plasma cells can begin to appear several days after inflammation occurs. After exposure to antigens that trigger the body's immune response, B cells activate, proliferate, and differentiate into plasma cells that produce antibodies. The role of plasma cells in the inflammatory process is to help eliminate pathogens and reduce inflammation by producing antibodies that will help bind and destroy pathogens. This process may take time, depending on the complexity of the infection and the ability of the individual's immune system to respond efficiently 22.

The consistency of these findings with previous research underscores the reliability of probiotics as a therapeutic option in wound care. Studies such as those by Tagliari *et al.* have demonstrated the benefits of probiotics in enhancing wound healing in animal models, particularly through mechanisms that reduce bacterial load and promote tissue regeneration 5. The current study confirms these benefits and provides new insights into how the mode of administration can influence therapeutic outcomes. This comparison with existing literature underscores the need for further research to explore the full potential of probiotics in different clinical scenarios.

In summary, this study offers significant contributions to understanding probiotics in wound healing, particularly highlighting the advantages of topical over systemic administration. The findings suggest that topical probiotics could be a more effective treatment modality, offering both localized immune modulation and enhanced tissue repair. Future research should focus on clinical trials to validate these findings in human subjects and explore the potential of various probiotic strains and formulations in wound care.

## Conclusion

This study demonstrates that topical administration of *L. casei Shirota* is more effective than systemic administration in enhancing the wound healing process, as evidenced by the increased presence of macrophages and plasma cells and the decreased expression of TNF-α. These findings suggest that topical probiotics may be a promising treatment strategy for improving wound healing, warranting further clinical investigation.

## Figures and Tables

**Figure 3:**
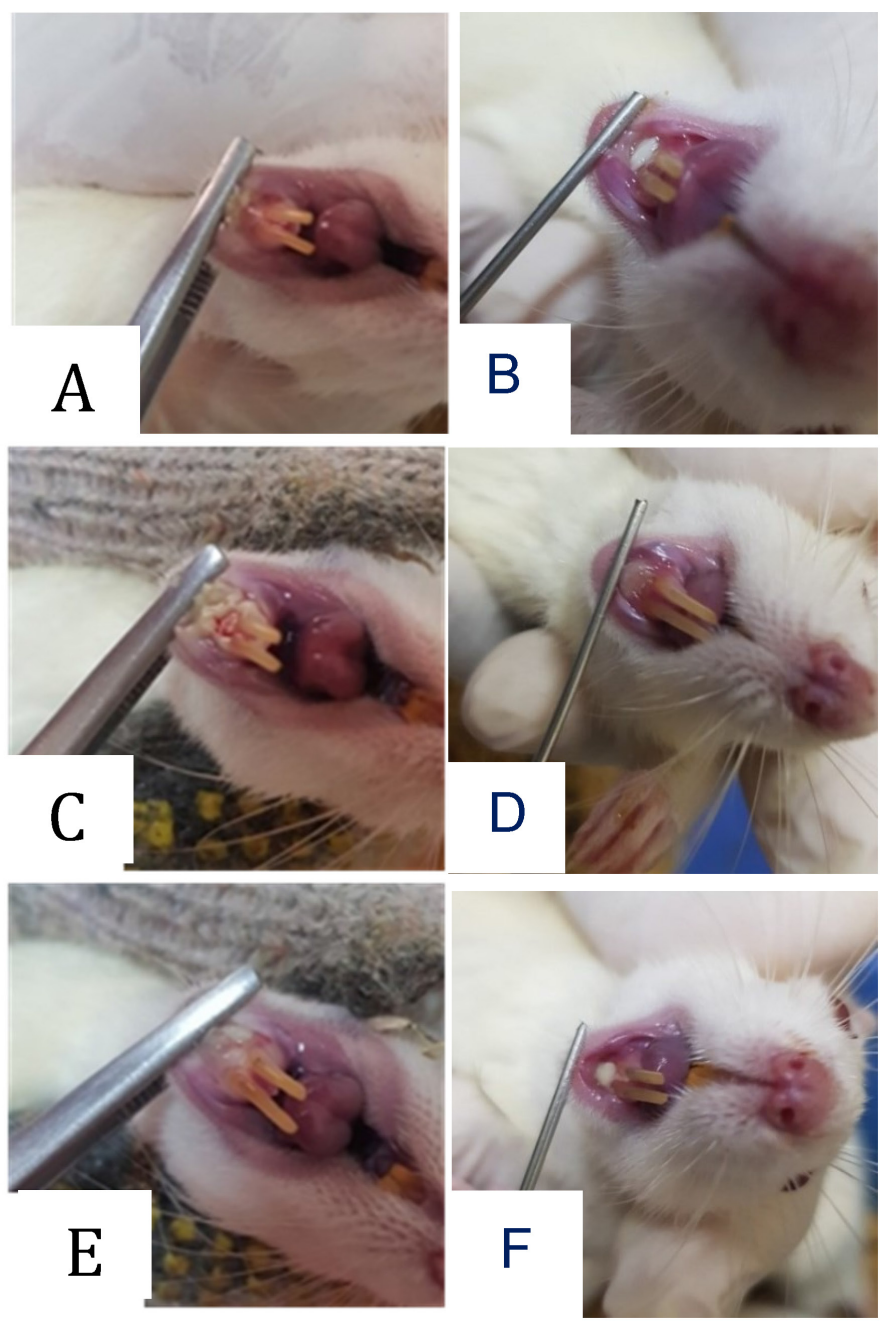
Clinical appearance of oral traumatic ulcer in the animal model. The traumatic ulcers were formed after 24-48 hours in the 3-day control group (A) and the 7-day control group (B). Systemic (C) and topical (D) administration probiotic treatment procedures were employed for 3 days. The recovery results were shown after 7 days by systemic (E) and topical (F) administration probiotic treatment procedures.
